# Brain functional activity of Parkinson’s disease patients under a virtual reality eye movement task: a functional near-infrared spectroscopy study

**DOI:** 10.3389/fnhum.2025.1624668

**Published:** 2025-12-02

**Authors:** Yanzhi Liu, Ziqian Shi, Lifeng Wang, Chuyan Yang, Xinyan Wang, Yongzhong Lin, Liping Qi

**Affiliations:** 1 Department of Neurology, The Second Hospital of Dalian Medical University, Dalian, China; 2 School of Computer Science and Technology, Dalian University of Technology, Dalian, China; 3 Department of Emergency Medicine, The First Affiliated Hospital of Dalian Medical University, Dalian, China; 4 School of Control Science and Engineering, Dalian University of Technology, Dalian, China

**Keywords:** Parkinson’s disease, PD, virtual reality, VR, eye movement, functional near-infrared spectroscopy, fNIRS

## Abstract

**Objective:**

This study aimed to investigate the eye movement behavior characteristics and associated brain functional activity changes in Parkinson’s disease (PD) patients during a complex visual task, using virtual reality (VR) eye movement tasks combined with functional near-infrared spectroscopy (fNIRS) technology.

**Methods:**

A total of 27 PD patients and 29 healthy controls were included in the study. Participants performed a “Whack-a-Mole” eye movement task on a VR platform. Fixation time and task error rates were recorded, and fNIRS was used to measure changes in brain oxygenation. The differences in oxygenated hemoglobin concentration in brain regions between PD patients and healthy controls were assessed during task performance.

**Results:**

The PD group exhibited a significantly higher task error rate compared to the control group (*p* = 0.02), and a significantly longer mean fixation time (*p* = 0.001). fNIRS results revealed that the PD group had considerably higher oxygenated hemoglobin concentrations in the bilateral primary visual cortex (V1), visual association cortex, primary somatosensory cortex (S1), and auditory cortex compared to the control group (*p* < 0.05).

**Conclusion:**

PD patients exhibit significant eye movement behavioral impairments during the execution of complex visual tasks, accompanied by compensatory brain functional activation in relevant brain regions. These findings provide important insights for the early diagnosis and therapeutic intervention of PD.

## 1 Introduction

Parkinson’s disease (PD) is characterized not only by motor symptoms but also by abnormalities in eye movement functions, such as delayed saccades and tracking impairments, which have been shown to be closely associated with the pathological progression of the disease (Xu et al., 2024; Lage et al., 2024; Beheshti et al., 2024). Therefore, the identification and monitoring of eye movement abnormalities are not only beneficial for the early diagnosis of PD but also provide potential biomarkers for assessing disease progression (Fooken et al., 2022). Although previous studies have revealed eye movement abnormalities in PD patients, the underlying neural mechanisms, particularly the functional activity changes in brain regions responsible for eye movement control, remain unclear (Antoniades and Spering, 2023). As eye movement is a complex motor control function that reflects the coordination of multiple neural circuits in the brain, investigating the eye movement characteristics of PD patients can contribute to a deeper understanding of the neural mechanisms behind their motor dysfunction.

Traditional eye movement detection methods, such as video-based eye trackers and electrooculography (EOG), despite being widely used in research, have several limitations. These techniques generally require participants to be in a static experimental environment, making it difficult to simulate dynamic, real-world visual scenarios, which limits the understanding of eye movement control in complex situations. To overcome these limitations, virtual reality (VR) technology has emerged as an innovative solution. VR can create highly immersive dynamic visual environments, allowing researchers to measure eye movement responses of PD patients in settings that are closer to real-world scenarios, thus revealing eye movement characteristics with greater ecological validity (Rodríguez-Mansilla et al., 2023; Madison et al., 2025). Additionally, VR technology can be combined with high-precision eye tracking to capture real-time ocular movements, providing richer data for investigating the relationship between eye movement behavior and perceptual-motor function (Xu et al., 2024). However, relying solely on eye movement data still cannot fully elucidate the functional changes in the associated neural networks in the brain.

To further explore the brain functional activity of PD patients during the execution of eye movement tasks, this study integrates functional near-infrared spectroscopy (fNIRS) technology. fNIRS can monitor real-time neural activity in specific brain regions by detecting changes in the concentration of oxygenated and deoxygenated hemoglobin (Pinti et al., 2020). Compared to other neuroimaging techniques, such as functional magnetic resonance imaging (fMRI), fNIRS offers higher temporal resolution and is less sensitive to motion artifacts, making it particularly suitable for investigating brain function in PD patients during dynamic tasks (Quaresima and Ferrari, 2019; Liampas et al., 2024). Especially in complex virtual environments, fNIRS can effectively capture changes in brain functional activity, thereby revealing neural activity associated with eye movement (Acuña et al., 2024).

Building on these technological advantages, this study combined VR and fNIRS to investigate the eye movement characteristics of PD patients and the underlying brain functional changes, providing new insights for the early diagnosis and personalized treatment of PD.

## 2 Data and methods

### 2.1 General information

This study included 27 PD patients (PD group) who were diagnosed at the Department of Neurology, The Second Affiliated Hospital of Dalian Medical University, between February 2023 and February 2024, as well as 29 age- and gender-matched healthy individuals as the control group. The diagnosis of PD was based on the clinical diagnostic criteria for PD established by the *Movement Disorder Society* (MDS) in 2015 (Leng et al., 2022), and the disease stages were classified according to the Hoehn-Yahr staging scale, ranging from stage I to stage III. Inclusion criteria for the PD group were as follows: (1) PD patients aged between 40 and 80 years; (2) No severe visual impairments (e.g., inability to clearly distinguish visual cues or inability to reach normal vision levels even with corrective lenses); (3) No history of other severe neurological or psychiatric disorders (e.g., vestibular system disorders, epilepsy, encephalitis, dementia, schizophrenia, or severe depression); (4) No use of medications within the past 3 months that could potentially interfere with the study results, such as antipsychotics or anticonvulsants. Inclusion criteria for the control group were as follows: participants should have no history of neurological or psychiatric disorders and normal vision, or corrected vision reaching normal levels. Exclusion criteria for both groups included: (1) Presence of severe cardiovascular diseases, respiratory diseases, or other serious systemic conditions that could affect participation in the experiment; (2) Pregnant or breastfeeding women; (3) Known allergic reactions or intolerance to the equipment or procedures used in the experiment; (4) Patients who had previously participated in similar studies, potentially causing data bias; (5) Patients unable to comply with the experimental requirements.

There were no marked differences between the two groups in basic demographic characteristics such as gender and age (*p* > 0.05; Table 1). All participants provided written informed consent prior to the experiment, and the study was approved by the Ethics Committee of The Second Affiliated Hospital of Dalian Medical University, ensuring that the experimental procedures adhered to the ethical standards outlined in the Declaration of Helsinki.

**Table 1:** Eye movement task subject information (* represents significant difference, *p* < 0.05).

Baseline characteristics	PD group *n* = 27 (Mean ± standard deviation)	Control group *n* = 29 (Mean ± standard deviation)	*p*
Age (years old)	67.88 ± 8.39	65.11 ± 6.67	0.33
Gender (Male/Female)	10/17	17/12	0.11
Educational level (year)	9.79 ± 3.05	10.46 ± 3.17	0.45
MMSE	24.42 ± 3.31	28.20 ± 1.69	<0.01*
MoCA	20.09 ± 5.14	24.62 ± 3.09	<0.01*
Hoehn Yahr grading	2.15 ± 0.94	-	-
UPDRS-III	24.94 ± 18.57	-	-

Hoehn and Yahr, PD Hoehn-Yahr Staging Scale; UPDRS-III, Unified PD Rating Scale (UPDRS) Part III.

### 2.2 Eye movement task: VR platform

The eye movement task in this experiment was conducted using VR equipment, with the task designed as a visual tracking task. Participants were required to fixate on and maintain visual tracking of a moving target within the virtual environment. The target moved randomly at varying speeds and directions to increase the complexity of the task. Specifically, the task was based on a “Whack-a-Mole” game on the VR platform. When a mole appeared on the screen, participants were instructed to fixate on it, and the mole would automatically disappear once the participant’s gaze was directed at it. Prior to the formal test, all participants underwent a 3-min familiarization practice to ensure they were comfortable with the task process and operations. The practice results were not included in the formal data analysis. The formal test consisted of 15 “Whack-a-Mole” game modules, each lasting 10.5 s, with a 10-s rest between modules. In each game module, the frequency of mole appearances was dynamically adjusted based on the participant’s performance. If a participant responded quickly and accurately in the initial modules, the mole appearance frequency was increased to make the task more challenging. Conversely, if the participant had slower response times or lower accuracy, the frequency of mole appearances was decreased accordingly. In each module, the mole appearance frequency ranged from 1 to 2 times per second, with the appearance interval fluctuating between 0.5 and 1 s. Additionally, as the game progressed, the speed and randomness of the moles’ movements gradually increased to further enhance task difficulty and maintain challenge. During the test, participants were required to quickly identify and fixate on the moles to make them disappear. The VR system integrated a high-precision eye tracker that recorded the participants’ eye movement trajectories, fixation point changes, and reaction times in real-time. This system offered high spatial and temporal resolution, ensuring the accuracy and reliability of the eye movement data. All experimental data were used to analyze the eye movement characteristics of PD patients and the differences compared to the healthy control group.

### 2.3 Brain functional imaging: near-infrared spectroscopy technology

**Figure 1:**
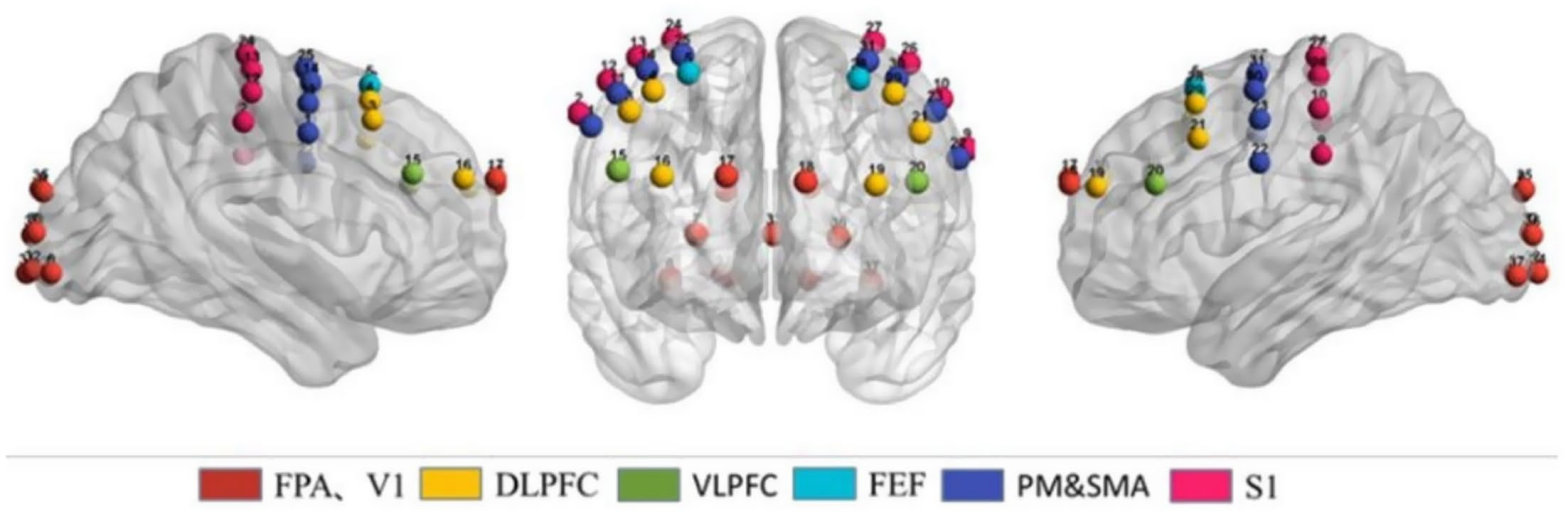
Channel layout of fNIRS equipment for eye movement experiment (FPA, DLPFC, VLPFC, FEF, PM&SMA, S1, and V1).

This study utilized the NirScan fNIRS brain function imaging device, manufactured by Danyang Huichuang Medical Equipment Co., Ltd., to collect near-infrared spectroscopy signals from the frontal, parietal, and occipital regions of the brain. The device consists of 26 optical fibers, with 14 serving as the light emission fibers and 12 as the light reception fibers. The linear distance between the emission and reception fibers is maintained at 3 cm, forming a total of 28 channels. The experiment used dual-wavelength (760 nm and 850 nm) near-infrared light for signal acquisition, with a sampling frequency of 10 Hz. Brain activity was recorded based on the estimation of near-infrared light absorption at both wavelengths for each channel and was converted into changes in the concentrations of oxygenated hemoglobin (HbO_2_) and deoxygenated hemoglobin (HbR) using the modified Beer–Lambert law. During the placement of the optical probe, the experiment continuously monitored and displayed the signal strength of each channel in real-time through the acquisition software to ensure data quality. The placement of the probes was based on the international “10–20” system. The most anterior probe was positioned around the FP1 and FP2 regions, the most lateral probes were placed in the PO7 and PO8 areas, the far left probe was near T3, and the far right probe was near T4. The primary measurement regions included the fronto-polar areas (Frontopolar Area, FPA) in both hemispheres, the dorsolateral prefrontal cortex (Dorsolateral Prefrontal Cortex, DLPFC), the ventrolateral prefrontal cortex (Ventrolateral Prefrontal Cortex, VLPFC), the frontal eye fields (Frontal Eye Fields, FEF), the pre-motor and supplementary motor cortex (Pre-Motor and Supplementary Motor Cortex, PM&SMA), the primary somatosensory cortex (Somatosensory Cortex, S1), and the primary visual cortex (Primary Visual Cortex, V1). With this setup, this study was able to accurately measure changes in brain activity in specific regions during the execution of the VR eye movement task by PD patients (Figure 1).

### 2.4 Data analysis

Eye movement behavioral data analysis was conducted. In this study, the “Whack-a-Mole” error rate and the average fixation time were selected as the primary indicators to assess participants’ eye movement abilities. The “Whack-a-Mole” error rate is defined as the percentage of moles that did not disappear after the participant fixated on them, relative to the total number of moles. This metric reflects the participant’s visual recognition and eye movement control abilities. The average fixation time is defined as the average time the participant fixates on the mole from its appearance to its disappearance, reflecting the participant’s eye movement speed and fixation stability. All behavioral data were statistically analyzed using *SPSS version 26.0*. First, the normality of the data was tested using the Shapiro–Wilk test. For data conforming to a normal distribution, two-sample t-tests were conducted to compare the differences in eye movement metrics between the PD group and the healthy control group. A significance level of *p* < 0.05 was set.

Brain function data analysis was conducted. The average blood oxygen changes in the task and resting states from the 15 task blocks were calculated by subtracting the baseline values. The baseline was defined as the average blood oxygen concentration during a 5-s period before the formal test, which served as the baseline level. The average blood oxygen changes during the task state, resting state, and the difference between the task and resting states were calculated. A two-sample t-test was performed on the blood oxygen changes in the task state, resting state, and task-resting state to compare the differences between the two groups. A significance level of *p* < 0.05 was set.

## 3 Results

### 3.1 Eye movement behavior

The task error rate was significantly higher in the PD group (35.62 ± 28.74%) than in the control group (19.45 ± 21.44%; *p* = 0.02). The average fixation time was also significantly longer in the PD group (880.62 ± 12.86 ms) than in the control group (742.98 ± 158.86 ms; *p* = 0.001; Table 2).

**Table 2:** The behavioral differences between the PD group and the control group in eye movement tasks (* represents a significant difference between the two groups of subjects, *p* < 0.05).

Parameters	PD group *n* = 27 (Mean ± standard deviation)	Control group *n* = 29 (Mean ± standard deviation)	*p*
“Whack-a-Mole” task error rate (%)	35.62 ± 28.74	19.45 ± 21.44	0.02*
Average fixation time (ms)	880.62 ± 12.86	742.98 ± 158.86	0.001*

### 3.2 Brain functional status under eye movement tasks

**Figure 2:**
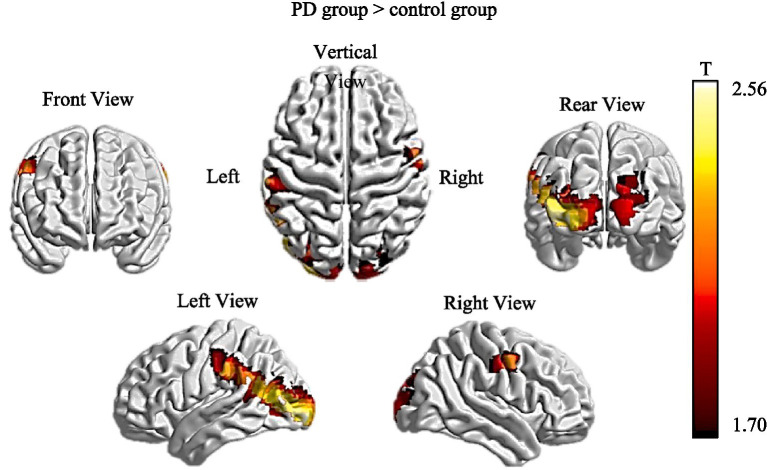
Differences in cortical activation between the PD group and the control group during the eye movement task.

The specific brain region activation differences are shown in Figure 2, which highlights significant activations in these regions (yellow and red areas in the figure). In the eye movement “Whack-a-Mole” task, changes in blood oxygen concentration between the task and resting states were calculated, and a two-sample t-test was used to analyze the differences in oxygenated hemoglobin concentration in brain regions between the PD group and the healthy control group. The results showed that the PD group had significantly higher concentrations of oxygenated hemoglobin in the bilateral primary visual cortex (V1), visual association cortex, primary somatosensory cortex (S1), and auditory cortex compared to the control group (*p* < 0.05).

(The statistical map (t-values) overlaid on a brain template shows brain regions with significantly higher oxygenated hemoglobin concentration in the PD group compared to the controls (*p* < 0.05). Yellow-to-red colors indicate higher activation in the PD group. Views from the top, front, left, right, and rear are displayed).

## 4 Discussion

This study used a VR platform combined with fNIRS to evaluate the behavioral performance and associated brain function changes of PD patients during an eye movement task. The results showed that PD patients had a significantly higher error rate and significantly longer average fixation time during the “Whack-a-Mole” task compared to the healthy control group. The performance differences in the visual tracking task suggest that eye movement control in PD patients is affected by their neurological condition, which is consistent with previous studies on eye movement abnormalities in PD patients. This delayed eye movement response may result from impairments in the basal ganglia-frontal lobe circuit in PD patients, thereby affecting their ability to quickly respond to visual stimuli (Ávila, 2024).

In terms of brain function imaging, fNIRS revealed significant activation in the bilateral V1, visual association cortex, S1, and auditory cortex in PD patients during task execution. However, no significant activation was observed in the FEF, which may be related to the degeneration of the frontal cortex and the dysregulation of associated networks in PD patients (Ji et al., 2023; Masilamoni et al., 2022). FEF are typically considered a key brain region for regulating visual tracking and eye movement, particularly playing a crucial role in rapid saccadic eye movements and target tracking tasks (Hanning et al., 2023; Qian et al., 2025). Studies noted that the basal ganglia-frontal lobe network in PD patients is severely affected during disease progression, leading to impairments in frontal lobe functions, including eye movement control (Revankar et al., 2020). This dysfunction of the frontal-basal ganglia network may explain the lack of significant activation in the FEF observed in our study.

Although no significant activation was observed in the FEF, PD patients exhibited significant activation in other brain regions, such as the V1, visual association cortex, S1, and auditory cortex. This increased brain activity suggests that PD patients may require more neural resources to process visual information and complete the same task, which aligns with their behavioral performance. The enhanced activity in the V1 and visual association cortex may be related to the higher cognitive load required by PD patients when performing visual tasks. Under normal conditions, these regions are primarily responsible for basic visual processing and integration. However, due to the decline in motor and cognitive functions in PD patients, they may become more reliant on visual perception to complete the task (Tong et al., 2025). Previous studies have also indicated that PD patients often exhibit additional visual cortex activation during complex visual tasks, potentially compensating for deficits in motor control functions (Nitschke et al., 2023).

Furthermore, the significant activation observed in the S1 and auditory cortex may be related to sensory integration issues in PD patients. Research has shown that PD patients not only exhibit motor control deficits but may also experience multisensory integration impairments (Permezel et al., 2023). This abnormal sensory processing could be one of the reasons why patients require more time to fixate on target objects and make more errors during the task. When the brain attempts to integrate visual, auditory, and sensory information, it encounters difficulties and must mobilize additional neural resources, leading to heightened brain activity. These findings support the hypothesis that PD patients require additional brain region activation to complete perceptual-motor tasks, especially those involving multisensory integration, such as visual, somatosensory, and auditory processing (Jordi and Isacson, 2024; Luft et al., 2020).

It is noteworthy that the significant activation observed in multiple brain regions of PD patients in this study may represent a compensatory mechanism. This is consistent with previous findings, which suggest that PD patients activate additional networks during complex tasks to compensate for deficiencies in the basal ganglia circuits, serving as a compensatory response to damage (Youn et al., 2023; Johansson et al., 2024). The activation patterns of these brain regions, particularly the abnormalities in the primary visual cortex, somatosensory cortex, and multisensory integration areas, could serve as early diagnostic biomarkers for PD. As the disease progresses and compensatory mechanisms change, alterations in brain region activation can be used to monitor disease deterioration. Furthermore, individualized treatment plans could be tailored based on the unique brain activation patterns of each patient, targeting the enhancement or modulation of specific brain regions to improve therapeutic outcomes.

As demonstrated above, PD patients exhibit considerable eye movement abnormalities during the execution of complex visual tasks, accompanied by compensatory brain function activation in relevant brain regions. These findings provide important insights for the early diagnosis and potential therapeutic intervention of PD. However, the limitations of this study include the relatively small sample size, which may impact the generalizability of the results. Future studies should aim to increase the sample size to verify the stability and broader applicability of these findings. Additionally, the limitations of the task design in this study may influence the interpretation of the results. Future research could consider designing more diverse tasks to comprehensively assess the brain function changes in PD patients.

## 5 Conclusion

This study demonstrates that PD patients exhibit significant eye movement abnormalities during the execution of VR eye movement tasks, accompanied by compensatory brain function activation in relevant brain regions. These findings provide new evidence for the early diagnosis and personalized treatment of PD. The results suggest that the combination of VR and fNIRS technology may have broad application prospects in this field.

## Data Availability

The original contributions presented in the study are included in the article/supplementary material, further inquiries can be directed to the corresponding authors.
